# Application of artificial intelligence to ultrasound imaging for benign gynecological disorders: systematic review

**DOI:** 10.1002/uog.29171

**Published:** 2025-01-31

**Authors:** F. Moro, M. T. Giudice, M. Ciancia, D. Zace, G. Baldassari, M. Vagni, H. E. Tran, G. Scambia, A. C. Testa

**Affiliations:** ^1^ Dipartimento Scienze della Salute della Donna, del Bambino e di Sanità Pubblica Fondazione Policlinico Universitario Agostino Gemelli, IRCCS Rome Italy; ^2^ UniCamillus International Medical University Rome Italy; ^3^ Infectious Disease Clinic, Department of Systems Medicine Tor Vergata University Rome Italy; ^4^ Radiomics G‐STeP Research Core Facility, Fondazione Policlinico Universitario A. Gemelli, IRCCS Rome Italy; ^5^ Istituto di Radiologia Università Cattolica del Sacro Cuore Rome Italy; ^6^ Dipartimento Universitario Scienze della Vita e Sanità Pubblica Università Cattolica del Sacro Cuore Rome Italy

**Keywords:** artificial intelligence, gynecology, machine learning, ultrasonography

## Abstract

**Objective:**

Although artificial intelligence (AI) is increasingly being applied to ultrasound imaging in gynecology, efforts to synthesize the available evidence have been inadequate. The aim of this systematic review was to summarize and evaluate the literature on the role of AI applied to ultrasound imaging in benign gynecological disorders.

**Methods:**

Web of Science, PubMed and Scopus databases were searched from inception until August 2024. Inclusion criteria were studies applying AI to ultrasound imaging in the diagnosis and management of benign gynecological disorders. Studies retrieved from the literature search were imported into Rayyan software and quality assessment was performed using the Quality Assessment Tool for Artificial Intelligence‐Centered Diagnostic Test Accuracy Studies (QUADAS‐AI).

**Results:**

Of the 59 studies included, 12 were on polycystic ovary syndrome (PCOS), 11 were on infertility and assisted reproductive technology, 11 were on benign ovarian pathology (i.e. ovarian cysts, ovarian torsion, premature ovarian failure), 10 were on endometrial or myometrial pathology, nine were on pelvic floor disorder and six were on endometriosis. China was the most highly represented country (22/59 (37.3%)). According to QUADAS‐AI, most studies were at high risk of bias for the subject selection domain (because the sample size, source or scanner model was not specified, data were not derived from open‐source datasets and/or imaging preprocessing was not performed) and the index test domain (AI models were not validated externally), and at low risk of bias for the reference standard domain (the reference standard classified the target condition correctly) and the workflow domain (the time between the index test and the reference standard was reasonable). Most studies (40/59) developed and internally validated AI classification models for distinguishing between normal and pathological cases (i.e. presence *vs* absence of PCOS, pelvic endometriosis, urinary incontinence, ovarian cyst or ovarian torsion), whereas 19/59 studies aimed to automatically segment or measure ovarian follicles, ovarian volume, endometrial thickness, uterine fibroids or pelvic floor structures.

**Conclusion:**

The published literature on AI applied to ultrasound in benign gynecological disorders is focused mainly on creating classification models to distinguish between normal and pathological cases, and on developing models to automatically segment or measure ovarian volume or follicles. © 2025 The Author(s). *Ultrasound in Obstetrics & Gynecology* published by John Wiley & Sons Ltd on behalf of International Society of Ultrasound in Obstetrics and Gynecology.

## INTRODUCTION

Ultrasound examination is the first‐line method for the diagnosis and management of many gynecological diseases^1^. In recent decades, ultrasound has played a fundamental role in the fields of endometriosis^2^, ^3^, ^4^, reproductive medicine^5^, ^6^, ^7^ and pelvic floor disorders. Advances in the quality and availability of transvaginal ultrasound machines have led to improvements in the diagnosis of deep endometriosis^8^, ^9^, ^10^, ^11^ and the monitoring of infertile women undergoing assisted reproductive technology (ART) (e.g. assessment of follicular maturity and endometrial factors affecting embryo implantation)^12^. Ultrasound is the imaging method of choice in the perioperative assessment of patients with pelvic floor dysfunction to evaluate the urethra, bladder neck, rectum and anorectal junction as well as the integrity of the pelvic muscles^13^.

The introduction of artificial intelligence (AI) in imaging is revolutionizing the diagnosis and management of gynecological disease. AI includes machine learning (ML) models, which are trained to recognize patterns and relationships from input data without explicit programming^14^, and deep learning (DL) models, a subset of ML using artificial neural networks with multiple layers (deep architectures) capable of learning complex representations from data. Convolutional neural networks (CNNs) are a subtype of DL models that can automatically learn spatial hierarchies of characteristics from input images^15^. CNNs are composed of multiple layers: convolutional layers for extracting image characteristics; pooling layers for reducing dimensionality; and fully connected layers for classification^16^. A detailed description of key concepts in AI is provided in Appendix S1.

ML and DL techniques are often employed in radiomics research to analyze and interpret large quantities of data generated from medical images. Radiomics is a process that extracts features from medical images and provides a quantitative description of the imaging data. The radiomics workflow involves the segmentation of the region of interest (ROI) from the studied image, image processing for subsequent analysis, extraction of radiomics features from the ROI and analysis of the extracted features to identify patterns, correlations and associations with clinical outcomes^17^.

There are several applications of AI in ultrasound imaging, including detection (i.e. the automatic identification of organ structures and other objects of interest), classification (i.e. the analysis of ultrasound images to assess disease status or classify pathology into a specific category) and segmentation (i.e. the delineation of precise lesion boundaries, such as those of ovarian follicles or cysts)^18^.

Many authors have explored AI in gynecology^19^, ^20^, ^21^, ^22^, ^23^, ^24^, ^25^, ^26^, ^27^, ^28^, ^29^, ^30^, ^31^, ^32^, ^33^, ^34^, ^35^, ^36^, ^37^, ^38^, ^39^, ^40^, ^41^, ^42^, ^43^, ^44^, ^45^, ^46^, ^47^, ^48^, ^49^, ^50^, ^51^, ^52^, ^53^, ^54^, ^55^, ^56^, ^57^, ^58^, ^59^, ^60^, ^61^, ^62^, ^63^, ^64^, ^65^, ^66^, ^67^, ^68^, ^69^, ^70^, ^71^, ^72^, ^73^, ^74^, ^75^, ^76^, ^77^, ^78^, ^79^, ^80^, ^81^, ^82^, ^83^, ^84^, ^85^, ^86^, ^87^, but there is a lack of synthesis of the available evidence regarding ultrasound‐based AI methods. The aim of this systematic review was to synthesize and evaluate the existing evidence on how AI technologies can enhance the accuracy, efficiency and predictive capability of ultrasound imaging for the diagnosis, management and monitoring of benign gynecological disorders, considering the strengths, limitations and potential gaps that may guide future research in this field.

## METHODS

This systematic review was conducted and reported according to the Preferred Reporting Items for Systematic reviews and Meta‐Analyses (PRISMA) statement. The protocol was registered with the PROSPERO database (CRD42023427088).

### Search strategy

A literature search was conducted in Web of Science, PubMed and Scopus to retrieve potentially eligible articles, published from inception until 4 August 2024. A search string for PubMed was constructed from medical subject headings (MeSH) terms, keywords and free‐text words, such as ‘radiomics’, ‘ultrasound‐based radiomics’, ‘artificial intelligence’, ‘machine learning’, ‘deep learning’, ‘ultrasonography’, ‘gynecology’, ‘gynecological diseases’, ‘endometrium’, ‘uterus’, ‘uterine’, ‘ovary’, ‘ovarian’, ‘ovaries’ and ‘fallopian tube’. The search was restricted to studies performed on humans and reported in the English language. No other restrictions were applied. The search string was adapted for use in the two other electronic databases. The full search strategy for all databases is provided in Appendix S2.

### Inclusion and exclusion criteria

We included articles reporting the role of AI applied to ultrasound in benign gynecological disorders, focusing specifically on diagnosis, outcome, image acquisition, segmentation, type of AI, model input and model performance. We excluded systematic reviews, non‐empirical studies, conference abstracts, editorials, commentaries, book reviews and abstracts not accompanied by a full‐text article. Furthermore, animal and modeling studies were excluded.

### Study selection

All studies retrieved from the literature search were imported to Rayyan software^88^ and duplicates were removed. Rayyan is a web‐based tool designed to facilitate the screening and selection of studies for systematic reviews^89^. We chose to use this tool because it improves efficiency, reduces bias and helps to organize documents and the decision‐making process, thus ensuring a more rigorous and transparent review.

Two researchers (F.M., M.T.G.) independently performed the first round of screening based on titles and abstracts. Discrepancies were resolved by discussion. Subsequently, studies with the full text available were read in their entirety by four researchers (F.M., M.T.G., M.C., G.B.) to select the final articles to include in the review and disagreements were resolved by discussion. If more than one study was published for the same cohort with identical endpoints, the report containing the most comprehensive information on the population was included to avoid overlapping populations. The reference lists of the included studies were searched manually for additional studies. When it was not possible to retrieve the full text online, we contacted the corresponding author of the article.

### Data extraction

Data extraction was performed by four researchers (F.M., M.T.G., M.V., G.B). A dedicated data extraction form was used to retrieve the following information for each eligible study: (1) study identifiers (first author, title, publication year); (2) study characteristics (study period, country, population, type of gynecological disorder); (3) objective of the study; (4) AI specifics (type of AI being assessed, model input); and (5) main findings and model performance.

Because a quantitative synthesis was not feasible because of the high heterogeneity of the included studies, resulting from differences in study design, population, interventions, outcomes and measurement methods, we produced a qualitative summary of the findings in the form of a narrative synthesis. This approach allowed us to systematically explore and describe these differences, providing a structured and comprehensive summary of the evidence that, despite the diversity of the data, offers valuable insights and a deeper understanding of the topic. The information retrieved from the included articles was categorized according to the type of AI assessed and the gynecological disease studied, and was structured using Excel spreadsheets (Microsoft, Redmond, WA, USA). The findings were summarized in a dedicated table, including the specific AI used, the study setting, the gynecological disease and the objective for each study.

For studies that developed multiple AI models, we reported the results for all models. Performance was reported as the area under the receiver‐operating‐characteristics curve (AUC) or, if unavailable, as accuracy, for studies aiming to discriminate between categories (e.g. presence *vs* absence of polycystic ovary syndrome (PCOS), pelvic floor disorder or pelvic endometriosis). For studies with the sole aim of automated segmentation (e.g. of the ovary or follicle), the Dice similarity coefficient (DICE) was reported. DICE is a statistical measure used to assess the similarity between two groups, such as the automated segmentation produced by an algorithm and the ground truth segmentation^90^. It is calculated as follows:

$$\text{DICE}=2\times \frac{\left|A\cap B\right|}{\left|A\right|+\left|B\right|}$$

where A and B represent the two segmentations being compared. The DICE value ranges from 0 to 1, with 1 indicating perfect agreement and 0 indicating no overlap. We chose to report the DICE value, among other metrics, because it is the measure used most widely to assess the quality of segmentation^88^. There are several other metrics that can be used to assess the performance of models. The structural similarity index is a perceptual metric used to measure the similarity between two given images, taking into account changes in structural information, luminance and contrast, and providing an assessment of image quality as perceived by human vision^91^. Its value ranges from −1, meaning that the compared images are very different, to 1, meaning that the compared images are the same. Missed segmentation ratio quantifies the proportion of pixels that the algorithm fails to detect or correctly segment^32^. It is computed as the ratio between the number of segmented pixels and the total number of pixels inside the ground truth segmentation. Its value ranges from 0, indicating perfect segmentation, to 1, indicating failed segmentation. The intraclass correlation coefficient is a statistical measure used to assess the consistency of measurements made by different raters; its value ranges from 0, indicating no agreement, to 1, indicating perfect agreement^92^. The F1 score is a metric used to evaluate the performance of a classification model, especially in scenarios in which the balance between precision and recall is crucial; it is given by the harmonic mean of precision and recall^88^. An F1 score of 1 indicates perfect precision and recall, while 0 indicates that either precision or recall is zero. The performance reported herein refers to the external validation set or, when absent, to the internal validation set; if no validation was performed, this was reported.

### Quality assessment

The overall quality of the included studies was assessed using the Quality Assessment Tool for Artificial Intelligence‐Centered Diagnostic Test Accuracy Studies (QUADAS‐AI)^93^. The specific criteria are listed in Table S1. This tool represents an extension and revision of QUADAS‐2^94^ and QUADAS‐C^95^ guidelines, and uses four domains (subject selection, index test, reference standard, workflow) to evaluate the risk of bias in AI‐centered studies.

## RESULTS

### Search strategy

**Figure 1:**
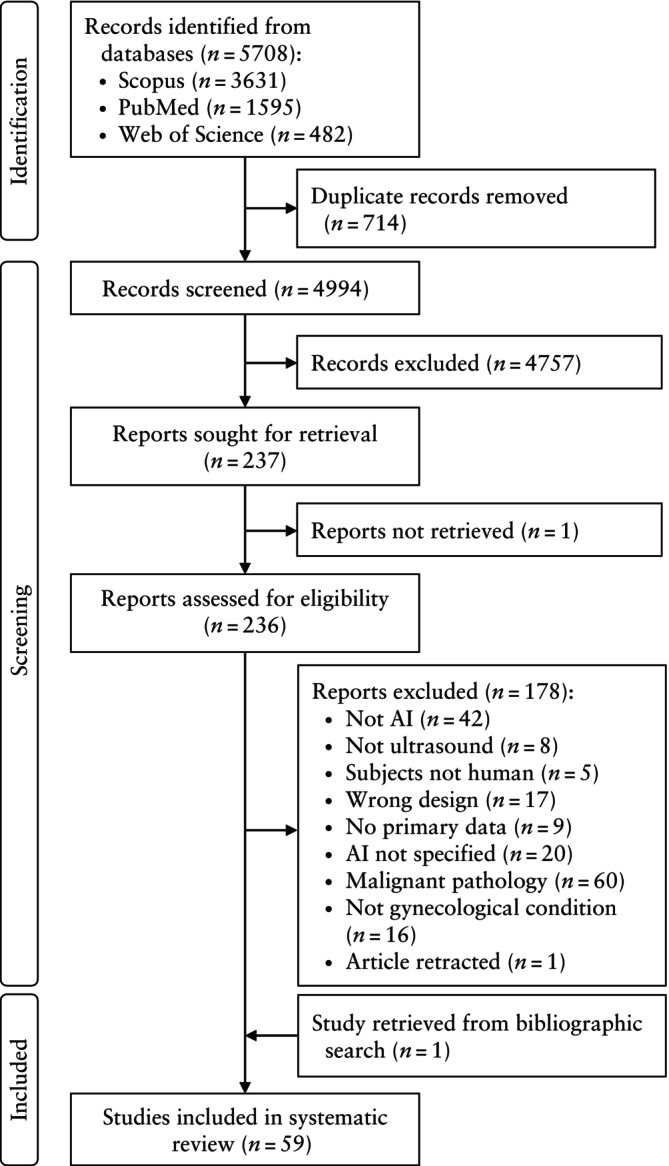
Flowchart summarizing inclusion of articles in systematic review. AI, artificial intelligence.

Our literature search retrieved a total of 4994 unique articles. After the screening and selection process, 59 studies reporting on benign gynecological conditions were accepted for inclusion in the systematic review^29^, ^30^, ^31^, ^32^, ^33^, ^34^, ^35^, ^36^, ^37^, ^38^, ^39^, ^40^, ^41^, ^42^, ^43^, ^44^, ^45^, ^46^, ^47^, ^48^, ^49^, ^50^, ^51^, ^52^, ^53^, ^54^, ^55^, ^56^, ^57^, ^58^, ^59^, ^60^, ^61^, ^62^, ^63^, ^64^, ^65^, ^66^, ^67^, ^68^, ^69^, ^70^, ^71^, ^72^, ^73^, ^74^, ^75^, ^76^, ^77^, ^78^, ^79^, ^80^, ^81^, ^82^, ^83^, ^84^, ^85^, ^86^, ^87^ (Figure 1). Of those, 12 were on PCOS, 11 were on infertility and ART, 11 were on benign ovarian pathology (i.e. ovarian cysts, ovarian torsion, premature ovarian failure), 10 were on endometrial or myometrial pathology, nine were on pelvic floor disorder and six were on endometriosis.

The results of the quality assessment of the included studies using the QUADAS‐AI tool are presented in Table S2. Most studies were at high risk of bias for the subject selection domain (because the sample size or source was not specified, data were not derived from open‐source datasets, imaging preprocessing was not performed and/or information on the scanner model used to acquire images was not specified) and the index test domain (the AI model was not tested in an external population in most studies). However, there was generally a low risk of bias for the reference standard domain (in most studies, the reference standard classified the target condition correctly) and the workflow domain (the time elapsed between the index test and the reference standard was generally reasonable).

### Characteristics of included studies

The characteristics of the 59 included studies are summarized in Table S3. China was the most highly represented country (22 (37.3%) studies)^30^, ^33^, ^37^, ^52^, ^55^, ^58^, ^59^, ^60^, ^61^, ^62^, ^65^, ^69^, ^72^, ^73^, ^74^, ^75^, ^76^, ^77^, ^78^, ^81^, ^82^, ^87^, followed by India (nine (15.3%) studies)^31^, ^36^, ^38^, ^46^, ^47^, ^48^, ^49^, ^83^, ^84^. The number of patients analyzed ranged between 16^29^ and 25 535^44^, although in 20 (33.9%) studies^31^, ^32^, ^34^, ^38^, ^40^, ^41^, ^43^, ^45^, ^46^, ^47^, ^48^, ^53^, ^56^, ^57^, ^58^, ^79^, ^80^, ^83^, ^84^, ^86^, only the number of images was specified.

Most papers (47 (79.7%))^3^, ^29^, ^30^, ^31^, ^36^, ^37^, ^39^, ^40^, ^42^, ^45^, ^46^, ^47^, ^48^, ^49^, ^51^, ^52^, ^53^, ^54^, ^55^, ^57^, ^58^, ^59^, ^60^, ^61^, ^62^, ^63^, ^65^, ^66^, ^67^, ^68^, ^69^, ^70^, ^71^, ^72^, ^73^, ^74^, ^76^, ^77^, ^78^, ^79^, ^80^, ^81^, ^82^, ^83^, ^84^, ^85^, ^86^ were published between 2021 and 2024; the oldest studies included in the present review were published in 2007^43^, ^75^. Most studies (33 (55.9%)) developed DL models, 22 (37.3%) developed ML models, and both ML and DL models were developed in four (6.8%) studies. The AI models were implemented using different programming languages, namely Python (*n* = 20), MATLAB (*n* = 10) and R (*n* = 5), or software applications, including SPSS (*n* = 2) and MedCalc (*n* = 1); for the remaining 21 studies, no information was provided. The model code was publicly available for three studies^68^, ^82^, ^86^, available on request for six and presented in pseudocode in four, while no information was available in the remaining 46 studies.

Regarding manufacturer details, most studies (33 (55.9%)) did not report the vendor of the machine used to obtain the ultrasound images. Of the studies that did report the scanner model, 19/26 (73.1%) used only one scanner while 7/26 (26.9%) used two or more scanners.

### Infertility and assisted reproductive technology

Most studies (6/11) on infertile women undergoing ART developed models to automatically segment or measure the ovarian follicle, measure ovarian volume or track follicle growth^31^, ^32^, ^33^, ^34^, ^36^, ^87^. These studies developed either DL models (*n* = 5)^31^, ^33^, ^34^, ^36^, ^87^ or ML models (*n* = 1)^32^, using two‐dimensional or three‐dimensional (3D) ultrasound images as the model input, and showed good‐to‐high performance in terms of DICE. Two studies developed models to predict hyper‐response during ART cycles and included both ultrasound and clinical features as the model input: the ML model in one study had an AUC of 0.91^35^ and the DL model in the other study had an AUC of 0.88^30^. One study developed a ML model to differentiate between a favorable and non‐favorable uterine cavity for embryo implantation using amplitude‐ and frequency‐based features and reported accuracy of 0.94^29^. One study developed a ML model to evaluate endometrial elasticity measured by shear‐wave elastography in patients with unexplained infertility and demonstrated an AUC of 0.89^37^. Finally, Boneš *et al*. collected 3D ultrasound images from women in the general population, infertile women and women with recurrent spontaneous miscarriage, and developed a DL model for segmentation and alignment of uterine shape, which achieved high performance in terms of DICE (0.90)^86^.

### Polycystic ovary syndrome

Twelve studies developed ML models (*n* = 7)^42^, ^43^, ^44^, ^46^, ^47^, ^48^, ^49^, DL models (*n* = 3)^38^, ^39^, ^41^ or both (*n* = 2)^40^, ^45^ to discriminate between PCOS and normal ovary/ovarian cysts on ultrasound examination. The models included one or more of the following feature families as the model input: shape, intensity, ultrasound, clinical, morphological, stereological, geometrical, statistical and textural. The model performance was reported in terms of accuracy in 11 studies (ranging from 0.82 to 0.99) and as an F1 score (0.76) in one study. The largest study, performed by Cheng and Mahalingaiah in 2019^44^, including 25 535 patients and 39 093 ultrasound examinations, reported accuracy of 0.98 to discriminate between PCOS and normal ovarian morphology.

### Endometriosis

Three studies developed DL models to detect deep endometriosis, of which one used ultrasound and clinical data as the model input^50^ and two used only ultrasound images as the model input^52^, ^53^, with an AUC of 0.82, accuracy of 0.97 and DICE value of 0.82, respectively. One study developed a DL model, using ultrasound videoclips as the model input, to detect indirect signs of pelvic endometriosis (i.e. sliding sign), with an AUC of 0.96^51^. Finally, two studies developed models to distinguish between endometrioma and other types of adnexal lesion: one used a ML model including textural features and reported an AUC of 1^54^, while the other used a DL model based on ultrasound images and reported an AUC of 0.99^55^.

### Ovarian pathology (ovarian cysts, ovarian torsion, premature ovarian failure)

Six studies developed models to discriminate between different types of ovarian cyst^56^, ^57^, ^58^, ^59^, ^60^, ^61^. Sohail et al.^56^ used an ML model including both clinical and ultrasound features to discriminate between three types of cyst, showing accuracy of 0.88. Narmatha *et al*.^57^ used a DL method to distinguish between seven types of ovarian lesion, reporting accuracy of 0.97. Fan *et al*.^58^ developed a DL model to discriminate between normal pelvic cyst, ovarian cyst and non‐pure ovarian cyst, with accuracy of 0.96. Two studies developed models to distinguish endometrioma from other lesions: Liu *et al*.^60^ used a ML model to differentiate endometrioma from ovarian dermoid cyst and reported accuracy of 0.99, whereas Miao *et al*.^61^ developed a DL model with an AUC of 0.90 to discriminate between endometrioma and mucinous cystadenoma. Finally, Li *et al*.^59^ focused on cyst segmentation and endometrioma classification using a hybrid DL/ML model: for the first task, the authors developed a model composed of Swin Transformer and UPerNet, which showed a DICE value of 0.85; for the second task, the model comprised ResNet50 and Vision Transformer (ViT) and showed accuracy of 0.91.

Four studies developed ML models including both clinical and ultrasound features to identify adnexal torsion^63^, ^64^, ^65^, ^66^. Otjen *et al*.^64^ analyzed pediatric patients treated surgically for ovarian torsion at a quaternary pediatric hospital over an 11‐year period. They built a decision tree to identify torsion based on multiple features from pelvic ultrasound, yielding an AUC of 0.96. Chen *et al*.^65^ analyzed pediatric patients who underwent surgical confirmation of ovarian torsion over a 5‐year period. They built a ML model to identify torsion based on clinical and sonographic features, showing an AUC of 0.87. Turki and Raml^66^ built a support vector machine (SVM) with a linear kernel to discriminate between ovarian torsion and acute appendicitis, with an AUC of 0.99. Atia *et al*.^63^ developed a multivariate logistic regression model to predict adnexal torsion in patients who underwent laparoscopy for suspected torsion. The predictive performance was fair, with an AUC of 0.75.

Finally, Yu and Qing^62^ developed an AI segmentation algorithm using color Doppler ultrasound images (with peak systolic velocity, end‐diastolic velocity, resistance index and pulsatility index) that showed clearly the functional status and hemodynamic characteristics of the ovaries, which was used to diagnose idiopathic premature ovarian failure.

### Pelvic floor disorder

Six studies developed AI models to identify pelvic floor disorders^69^, ^70^, ^72^, ^73^, ^74^, ^75^. Of those, two studies developed AI models to predict stress urinary incontinence: one used a ML model including both clinical and ultrasound features and reported an AUC of 0.81^74^, and the other used a DL model including only ultrasound features and reported an AUC of 0.94^75^. The remaining four studies^69^, ^70^, ^72^, ^73^ developed AI models including ultrasound images, video footage or clinical and ultrasound features in patients with prolapse. For example, Duan *et al*.^73^ developed a DL algorithm including 3D ultrasound images as the model input to identify different types of pelvic organ prolapse (anterior *vs* middle *vs* posterior), reporting an AUC of 0.79. Xu *et al*.^72^ built a predictive ML model including clinical and ultrasound features to select the type of pessary (ring *vs* Gellhorn) for women with symptomatic prolapse, showing a AUC of 0.81.

Three studies focused on the identification and analysis of the anatomical structures involved in pelvic floor disorders^67^, ^68^, ^71^. Of those, two developed DL models, with DICE values of 0.79^67^ and 0.86^68^, respectively. One study^71^ developed a ML model with an AUC of 0.91.

### Uterine pathology

Two different DL models have been proposed for the assessment of endometrial thickness and endometrial adhesions using 3D ultrasound images^76^, ^77^. Wu and Zhang^77^ used an extreme learning machine denoising algorithm for the diagnosis of adhesions and found that it performed similarly to hysteroscopy. Wang *et al*.^76^ applied a 3D U‐Net model to 3D ultrasound images to perform automatic segmentation of the endometrium and recognition of endometrial adhesions, showing high segmentation accuracy (DICE, 0.91).

Seven studies developed models for the detection of fibroids in the uterus, using a DL approach (*n* = 6)^78^, ^79^, ^80^, ^81^, ^82^, ^84^ or a combined DL/ML method (*n* = 1)^83^. Dilna *et al*.^79^ used an ultrasound‐based DL model to detect the presence of fibroids, with accuracy of 0.95. Huo *et al*.^78^ analyzed 3870 ultrasound images from 667 patients using a deep convolutional neural network (DCNN), which performed well (AUC, 0.95). Shahzad *et al*.^80^ tested a dual‐path DCNN architecture on 1057 ultrasound images and reported accuracy of 0.998 in automatic fibroid detection, whereas Yang *et al*.^82^ showed a mean average precision of 0.98 for their DL model in a cohort of 871 patients. Cai *et al*.^81^ developed a hybrid DL model composed of MobileNetV2 and a deep convolutional generative adversarial network for the automatic detection of uterine fibroids, with accuracy of 0.97. The convolutional recurrent neural network of Chinna and Pathrose Mary^84^ had accuracy of 0.998 in detecting fibroids in uterine ultrasound images. Finally, Kaveramma *et al*.^83^ aimed to classify ultrasound images of the uterus as fibrotic or normal using ML and DL classifiers. Among ML classifiers, SVM produced a classification accuracy of 0.93, whereas that for ViT was 0.98.

Raimondo *et al*.^85^ evaluated the performance of a DL model to diagnose adenomyosis on uterine ultrasonographic images and the accuracy was low (0.51).

## DISCUSSION

In the present systematic review, we summarized the findings of studies that applied AI to ultrasound imaging in benign gynecological disorders. Most studies created classification models for distinguishing between normal and pathological cases (e.g. presence *vs* absence of PCOS, endometriosis and stress urinary incontinence). Others developed models to automatically segment or measure ovarian follicles or ovarian volume, with the aim of facilitating improved surveillance of the ovarian cycle, thereby optimizing ART in infertile women. Some limitations were identified in the included studies. For example, most did not perform external validation, which is a critical step in determining the reproducibility and generalizability of a prediction model to new and varied patient settings. In addition, the number and type of variables used for modeling varied widely between studies, making it difficult to compare models and assess model relevance. In addition, most studies were conducted in a single center, resulting in a limited number of cases with poor representation of patient characteristics. In addition, AI models were often trained on specific datasets, which can introduce bias, especially if the training data do not adequately represent diverse patient populations.

Several systematic reviews have been published on the use of AI in gynecological malignancy, presenting the principal findings and most common applications of AI to various imaging modalities^96^, ^97^, ^98^. The few systematic reviews that have been published on AI and ultrasound in the context of benign gynecological disorders have focused on a single condition^99^, ^100^. Avery *et al*.^99^ summarized the role of modern diagnostic techniques (i.e. transvaginal ultrasound examination, combined ultrasound and magnetic resonance imaging, and AI) in endometriosis. Of the 49 studies included, only three were on ultrasound imaging and AI. In their systematic review, Barrera *et al*.^100^ included 31 studies assessing the performance of ML algorithms in the diagnosis or classification of PCOS. Fourteen of these studies used ultrasound imaging, of which six were included in the present review; we excluded the remaining eight studies because they were reported in databases not included in our search strategy or the full text was not available.

Our review demonstrates that AI models are useful in several benign gynecological conditions, including endometriosis, endocrine disorders and pelvic floor dysfunction. Endometriosis and endocrine disorders are very common among women of reproductive age. Transvaginal ultrasound examination is usually the first‐line approach and is an excellent diagnostic tool when performed by experts. However, its diagnostic accuracy depends on operator experience level, ultrasound equipment and scanning time. AI models proved to be as accurate as an expert operator and therefore can be used in settings where expert operators are not available. We also found some applications of AI in pelvic floor disorders for which diagnosis using ultrasound can be difficult. The potential to predict pelvic floor disorder would be of great value in the preventive care of patients at highest risk, namely menopausal or postpartum women.

To the best of our knowledge, this is the first systematic review dedicated specifically to AI applied to ultrasound across the spectrum of benign gynecological disorders. We conducted an extensive literature search in multiple databases to ensure the rigor of the study. We reported comprehensive data including sample size, number of images, year of publication, geographical distribution and outcomes, as well as the type of AI and the families of variables included. Moreover, we assessed the quality of studies using QUADAS‐AI^93^, which is specifically adapted for AI research. However, some limitations should be acknowledged. First, the significant heterogeneity of the studies, in terms of AI algorithms, ultrasound techniques, patient populations and outcomes, prevented us from performing a meta‐analysis, which limited our ability to generalize the findings. In addition, rapid progress in the field of AI may diminish the long‐term relevance of these findings. Notable gaps in the literature include the lack of longitudinal studies evaluating the long‐term efficacy and safety of AI applications in ultrasound imaging for benign gynecological conditions, as well as a lack of studies addressing the practical challenges, barriers to clinical integration and ethical considerations associated with these technologies.

We believe that the present review can help readers to better understand the role of AI applied to ultrasound imaging. Incorporating AI systems into clinical practice can improve patient management and prognosis, reduce healthcare costs and reduce gynecologists' workload by increasing their efficiency and accuracy. Despite the advantages and developments described herein, the clinical translation of AI in gynecological diagnosis warrants further research and is a long‐term process.

In conclusion, the published literature on AI applied to ultrasound in benign gynecological disorders is focused mainly on creating classification models to distinguish between normal and pathological cases, and on developing models to automatically segment or measure ovarian follicles or volume. The present review should help readers to better understand the applications of AI to gynecological ultrasound imaging.

## Supporting information


**Appendix S1** Key concepts in artificial intelligence
**Appendix S2** Search strategy
**Table S1** Description of quality assessment based on QUADAS‐AI domains
**Table S2** Risk‐of‐bias assessment for 59 studies included in systematic review
**Table S3** Characteristics and key findings of 59 studies included in systematic review

## Data Availability

Data available on request from the authors.
