# Supplementary material for: Application of artificial intelligence to ultrasound imaging for benign gynecological disorders: systematic review
Source: Ultrasound Obstet Gynecol. 2025 Jan 31;65(3):295–302. doi: 10.1002/uog.29171 (PMC11872345; doi:10.1002/uog.29171)
Supplement: Supplementary file 1 — Appendix S1 Key concepts in artificial intelligence Appendix S2 Search strategy Table S1 Description of quality assessment based on QUADAS‐AI domains Table S2 Risk‐of‐bias assessment for 59 studies included in systematic review Table S3 Characteristics and key findings of 59 studies included in systematic review [file UOG-65-295-s001.docx]

**Supplementary material**

**Appendix S1:** Key concepts in artificial intelligence

**Artificial Intelligence** (AI) refers to the theory and development of computer systems able to perform tasks typically requiring human intelligence, such as recognizing patterns, making decisions, and solving problems. *[definition adapted from Oxford Languages Dictionary]* In medical imaging, AI systems help interpret images, detect anomalies, and predict clinical outcomes, enhancing diagnostic accuracy and treatment planning.

**Machine Learning** (ML) is the part of AI focusing on developing algorithms that learn from data to identify patterns and make predictions. ML models improve as they process more data through a learning process involving training, validation, and testing.

ML techniques can be classified into:

- **Supervised Learning**: models are trained on labeled data to predict outcomes based on input features, commonly used in classification tasks like distinguishing between benign and malignant masses in ultrasound images.
- **Unsupervised Learning**: models find patterns in unlabeled data, often used for clustering or identifying inherent groupings without predefined labels.

**Deep Learning** (DL) is a subset of ML that uses large multi-layer neural networks to model high-level abstractions in data. DL is particularly effective for analyzing images because it can automatically learn hierarchical features directly from raw data.

A **neural network** is a computational model, inspired by the brain, consisting of interconnected layers of neurons (nodes) that collectively model complex relationships in data. The input of a neural network can be of different nature and dimension. In case of neural networks processing images, they can accept as input both 2D and 3D images.

The neural networks can be used in two different learning ways:

- **End-to-end classification**: the neural network takes raw input data (e.g., an ultrasound image) and directly outputs a classification (e.g., benign or malignant). The network automatically learns the relevant features from the data without the need for manual feature extraction.
- **Feature Extraction**: the network processes the input image to extract high-level features, which can then be fed into other ML models for further analysis or classification. This approach allows for the combination of DL's powerful feature extraction capabilities with traditional ML techniques.

AI in medical imaging finds mainly application in the fields of classification and automatic segmentation:

- **Classification** involves analyzing images to determine the status or category of a disease. For example, a classification model might be trained to distinguish between different types of ovarian cysts (e.g., benign vs. malignant). Classification tasks typically result in a label or category being assigned to the entire image or specific regions within the image.
- **Automatic segmentation** refers to the process of delineating precise boundaries of anatomical structures or lesions within an image, such as outlining the shape of an ovarian follicle or cyst. Segmentation is crucial for accurate measurement, assessment, and treatment planning, as it provides detailed information about the size, shape, and location of structures. It can be used also to automatize the process of classification, by automating the Region Of Interest definition by a clinician.

The studies included in this review employ various ML and DL algorithms, each with unique strengths suited to different tasks in ultrasound imaging. Below we report a list of the most utilized ones.

- **k-Nearest Neighbor (kNN)**: a supervised, instance-based learning algorithm used for classification or regression tasks in ML. It works by finding the "k" closest data points (neighbors) to a given data point and then making predictions based on the majority class (for classification) or the average (for regression) of those neighbors in the feature space. The closeness of the neighbors is typically determined using distance metrics like the Euclidean distance. *[Fix, Evelyn; Hodges, Joseph L. (1951). Discriminatory Analysis. Nonparametric Discrimination: Consistency Properties (PDF) (Report). USAF School of Aviation Medicine, Randolph Field, Texas. Archived (PDF) from the original on September 26, 2020.] [Cover, Thomas M.; Hart, Peter E. (1967). "Nearest neighbor pattern classification" (PDF). IEEE Transactions on Information Theory. 13 (1): 21–27. CiteSeerX 10.1.1.68.2616. doi:10.1109/TIT.1967.1053964. S2CID 5246200.]*
- **Gaussian Mixture Models (GMMs)**: a probabilistic model commonly used for clustering in unsupervised learning. It assumes that data is generated from a combination of multiple Gaussian distribution, each with unknown parameters. In this context, each Gaussian distribution can represent a different cluster. The model uses the Expectation-Maximization (EM) algorithm to estimate the parameters of these distributions and to calculate the probability that each data point belongs to a specific cluster. For example, GMMs can be applied to group similar regions within an ultrasound image without prior labels. *[Reynolds, D. (2009). Gaussian Mixture Models. In: Li, S.Z., Jain, A. (eds) Encyclopedia of Biometrics. Springer, Boston, MA. https://doi.org/10.1007/978-0-387-73003-5_196]*
- **Logistic regression**: a statistical logistic model used for classification and predictive analytics. Logistic regression estimates the probability of an event occurring based on a given dataset of independent variables. In medical imaging, it might be used to predict the likelihood of a disease based on extracted features from ultrasound images. *[Hosmer, David W.; Lemeshow, Stanley (2000). Applied Logistic Regression (2nd ed.). Wiley.]*
- **Random Forest (RF)**: an ensemble learning method used for classification and regression tasks. It builds multiple decision trees during training and outputs the mode of the classes (for classification) or the average prediction (for regression) of the individual trees. By combining the predictions of multiple trees, RF improves accuracy and reduce the risk of overfitting compared to a single decision tree. *[Breiman, L. Random Forests. Machine Learning 45, 5–32 (2001). https://doi.org/10.1023/A:1010933404324]*
- **Support Vector Machine (SVM)**: a supervised learning algorithm used for classification and regression tasks. It finds the optimal hyperplane separating data points of different classes in the feature space. SVM aims to maximize the margin (distance) between the closest points of the classes (support vectors) and the hyperplane itself, ensuring that the model is both accurate and generalizes well to new data. *[Cortes, Corinna; Vapnik, Vladimir (1995). "Support-vector networks" (PDF). Machine Learning. 20 (3): 273–297. CiteSeerX 10.1.1.15.9362. doi:10.1007/BF00994018. S2CID 206787478.] [Vapnik, Vladimir N. (1997). "The Support Vector method". In Gerstner, Wulfram; Germond, Alain; Hasler, Martin; Nicoud, Jean-Daniel (eds.). Artificial Neural Networks — ICANN'97. Lecture Notes in Computer Science. Vol. 1327. Berlin, Heidelberg: Springer. pp. 261–271. doi:10.1007/BFb0020166. ISBN 978-3-540-69620-9.]*
- **Extreme Gradient Boosting (XGBoost)**: ML algorithm based on the gradient boosting framework. It builds an ensemble of multiple weak learners (often decision trees) sequentially, where each new tree attempts to correct the errors made by the previous ones to form a stronger classifier. XGBoost is known for its speed and performance, offering features like regularization to prevent overfitting and parallel processing to accelerate training. *[Tianqi Chen and Carlos Guestrin. 2016. XGBoost: A Scalable Tree Boosting System. In Proceedings of the 22nd ACM SIGKDD International Conference on Knowledge Discovery and Data Mining (KDD '16). Association for Computing Machinery, New York, NY, USA, 785–794. https://doi.org/10.1145/2939672.2939785]*
- **Convolutional Neural Network (CNN)**: it is a type of DL model specifically designed for processing structured grid data, such as images. CNNs utilize convolutional layers, pooling layers, and fully connected layers to automatically and hierarchically learn spatial hierarchies of features from input data. The convolutional layers apply filters to input data to extract features, while pooling layers reduce the dimensionality of the data, making CNNs particularly effective in image recognition and classification tasks. *[LeCun, Y., Bengio, Y. & Hinton, G. Deep learning. Nature 521, 436–444 (2015). https://doi.org/10.1038/nature14539]*
- **U-Net**: A CNN architecture designed specifically for image segmentation. It is widely used for tasks requiring precise delineation of structures within medical images, such as segmenting ovarian cysts or other gynecologic structures. *[Ronneberger, O., Fischer, P., Brox, T. (2015). U-Net: Convolutional Networks for Biomedical Image Segmentation. In: Navab, N., Hornegger, J., Wells, W., Frangi, A. (eds) Medical Image Computing and Computer-Assisted Intervention – MICCAI 2015. MICCAI 2015. Lecture Notes in Computer Science(), vol 9351. Springer, Cham. https://doi.org/10.1007/978-3-319-24574-4_28]*
- **Residual Network (ResNet)**: is a type of deep neural network that uses skip connections, or “residuals”, enabling the model to learn residual functions instead of direct mappings. This architecture helps in training very deep networks. *[K. He, X. Zhang, S. Ren and J. Sun, "Deep Residual Learning for Image Recognition," 2016 IEEE Conference on Computer Vision and Pattern Recognition (CVPR), Las Vegas, NV, USA, 2016, pp. 770-778, doi: 10.1109/CVPR.2016.90.]*

**Appendix S2.** Search strategy

(radiomics OR “ultrasound-based radiomics” OR “artificial intelligence” OR “ultrasound radiomics” OR “machine learning” OR “deep learning” OR “computer-aidedsegmentation” OR nomogram OR “deep neural network” OR “radiomic analysis” OR “radiomic” OR “computer-aided diagnosis” OR “transfer learning” OR “predictivemodel”) AND ("Ultrasonography"[Mesh] OR ultrasound OR "Ultrasonic Tomography" OR "Computer Echotomography" OR "Ultrasonic Diagnosis" OR Echography OR "Ultrasonographic Imaging" OR "Ultrasound Imaging" OR "Diagnostic Ultrasound" OR Echotomography OR Sonography) AND (gynecology [MESH] OR “gynecological diseases” endometrium OR uterus OR uterin OR ovary OR ovarian OR ovaries OR “fallopian tube” OR “polycystic ovary syndrome” OR PCOS OR vaginal OR vulvar OR “Endometrial cancer” OR “cervical cancer” OR “ovarian cancer” OR “ovarian neoplasm” OR “ovarian mass” OR “adnexa” OR “adnexal mass” OR “gynecological tumor” OR “lymph nodes” OR “ovarian tumor” OR “endometrial tumor” OR “cervical tumor” OR “cervix” OR “vulvar cancer”)

**Table S1.** Description of quality assessment based on QUADAS-AI domains

| **Domains** | **Subject selection** | **Index test (AI)** | **Reference standard** | **Workflow** |
| --- | --- | --- | --- | --- |
| Concerns | - Accurately characterize the source, size and quality of input data alongside clear patient eligibility criteria - Was it derived from open-source datasets? - Present the rationale and breakdown of its training, validation and test sets? - Whether to perform image pre-processing? - Provide the scanner model information used to acquire imaging data? | - Was external verification performed? | - Was the reference standard likely to correctly classify the target condition? | - Was the time between the index test and the reference standard reasonable? |

Summary of QUADAS-AI four domains with their concerns respectively clarified.

Risk of bias is judged as “low”, “high”, or “uncertain”.

1. If all questions for a domain are answered “yes” then risk of bias can be judged “low”.
2. If any question is answered “no” this marks the potential for bias: reviewers need to discuss to judge risk of bias.
3. The “uncertain” category should be used only when insufficient data are reported to permit a judgment.

**Table S2.** Risk-of-bias assessment for 59 studies included in systematic review

|  | **Risk of bias** | | | |
| --- | --- | --- | --- | --- |
|  | **Subject selection** | **Index test** | **Reference standard** | **Work flow** |
| Sammali et al, 2021 | **High** | **High** | **Low** | **Low** |
| Liang et al, 2022 | **High** | **High** | **Low** | **Low** |
| Gupta et al, 2021 | **High** | **High** | **Low** | **Low** |
| Chen et al, 2009 | **High** | **High** | **Low** | **Low** |
| Li et al, 2019 | **High** | **High** | **Unclear** | **Low** |
| Carvalho et al, 2019 | **High** | **High** | **Unclear** | **Low** |
| Robertson et al, 2020 | **High** | **High** | **Low** | **Low** |
| Srivastava et al, 2021 | **High** | **High** | **Low** | **Low** |
| Liang et al, 2020 | **High** | **High** | **Low** | **Low** |
| Li et al, 2024 | **High** | **High** | **Unclear** | **Low** |
| Bones et al, 2024 | **High** | **High** | **Unclear** | **Low** |
| Kumar, 2014 | **Unclear** | **High** | **Low** | **Low** |
| Alamoudi et al, 2023 | **High** | **High** | **Low** | **Low** |
| Suha et al, 2022 | **High** | **High** | **Low** | **Low** |
| Cahyono et al, 2017 | **High** | **High** | **Low** | **Low** |
| Madhumitha et al, 2021 | **High** | **High** | **Low** | **Low** |
| Lawrence et al, 2007 | **High** | **High** | **Low** | **Low** |
| Cheng et al, 2019 | **High** | **High** | **Unclear** | **Low** |
| Sumathi et al, 2023 | **High** | **High** | **Unclear** | **Low** |
| Jeevitha et al, 2022 | **High** | **High** | **Low** | **Low** |
| Gopalakrishnan et al, 2021 | **High** | **High** | **Low** | **Low** |
| Suganya et al, 2022 | **High** | **High** | **Low** | **Low** |
| Kiruthika et al, 2023 | **High** | **High** | **Low** | **Low** |
| Guerriero et al, 2021 | **High** | **High** | **Low** | **Low** |
| Maicas et al, 2021 | **High** | **Low** | **Low** | **Low** |
| Yang et al, 2021 | **High** | **High** | **Low** | **Low** |
| Podda et al, 2024 | **High** | **High** | **Low** | **Low** |
| Stefan et al, 2021 | **High** | **High** | **Low** | **Low** |
| Hu et al, 2023 | **High** | **High** | **Low** | **Low** |
| Sohail et al, 2010 | **High** | **High** | **Low** | **Low** |
| Narmatha et al, 2023 | **High** | **High** | **Low** | **Low** |
| Fan et al, 2023 | **High** | **High** | **Unclear** | **Low** |
| Li et al, 2024 | **High** | **High** | **Low** | **Low** |
| Liu et al, 2024 | **High** | **High** | **Low** | **Low** |
| Miao et al, 2024 | **High** | **High** | **Low** | **Low** |
| Yu et al, 2022 | **High** | **High** | **Low** | **Low** |
| Atia et al, 2023 | **High** | **High** | **Low** | **Low** |
| Otjen et al, 2020 | **High** | **High** | **Low** | **Low** |
| Chen et al, 2024 | **High** | **High** | **Low** | **Low** |
| Turki et al, 2023 | **High** | **High** | **Low** | **Low** |
| Garcia-Mejido et al, 2024 | **High** | **High** | **Low** | **Low** |
| Rabbat et al, 2023 | **High** | **High** | **Low** | **Low** |
| Zhu et al, 2022 | **High** | **High** | **Low** | **Low** |
| Van den Noort et al, 2023 | **High** | **High** | **Low** | **Low** |
| Muta et al, 2024 | **High** | **High** | **Low** | **Low** |
| Xu et al, 2021 | **High** | **High** | **Low** | **Low** |
| Duan et al, 2021 | **High** | **High** | **Low** | **Low** |
| Liu et al, 2022 | **High** | **High** | **Low** | **Low** |
| Huang et al, 2007 | **High** | **High** | **Low** | **Low** |
| Wang et al, 2022 | **High** | **High** | **Low** | **Low** |
| Wu et al, 2021 | **High** | **High** | **Unclear** | **Low** |
| Huo et al, 2023 | **High** | **Low** | **Low** | **Low** |
| Dilna et al, 2022 | **High** | **High** | **Low** | **Low** |
| Shahzad et al, 2023 | **High** | **High** | **Unclear** | **Low** |
| Cai et al, 2024 | **High** | **High** | **Low** | **Low** |
| Yang et al, 2023 | **High** | **High** | **Low** | **Low** |
| Kaveramma et al, 2023 | **High** | **High** | **Low** | **Low** |
| Chinna et al, 2024 | **High** | **High** | **Unclear** | **Low** |
| Raimondo et al, 2023 | **High** | **High** | **Unclear** | **Low** |

Summary of bias risk for each study included in the paper according to the QUADAS-AI domains. “High” = high risk of bias; “Low” = low risk of bias; “Uncertain” = insufficient data to permit the definition of bias whether high or low.

**Table S3**. Characteristics and key findings of 59 studies included in systematic review

| **Author, year, ref** | **Country** | **Time of assessment** | **N° patients**  **(n° images)** | **Gynecological disorder** | **Outcome** | **Type of AI (ML/DL)** | **Model's Input** ^§^ | **Best performing AI model** | **Performance of the best performing model** | **All other AI models** | **Performance of all other performing models** | **Type of validation** |
| --- | --- | --- | --- | --- | --- | --- | --- | --- | --- | --- | --- | --- |
| Sammali F  2021^29^ | Belgium | NA | 16 (NA) | Infertility (ART) | Classification of uterine activity as favorable or adverse to embryo implantation to predict pregnancy | ML | Amplitude and frequency-based features | K-nearest neighbors (KNN) | accuracy = 0.94  (in three considered measurement phases) | *Support Vector Machine (SVM)  *Gaussian Mixture Model (GMM) | SVM:  accuracy = 0.94 (in two of three measurement phases)  GMM:   accuracy = 0.88 (in one of three measurement phases) | internal |
| Liang X  2022^30^ | China | 2019-2020 | 181 (NA) | Infertility (ART) | Prediction of hyper-response (number of follicles) | DL | 2D-3D ultrasound images and clinical features (Estradiol level) | Multi-layer perception   multivariate classifier | AUC = 0.88 | * Decision Tree (DT)  * Support Vector Machine (SVM)  * k-nearest neighbours (KNN)  *Random Forest (RF) | DT: AUC =0.70 SVM:  AUC = 0.79  KNN: AUC= 0.73  RF: AUC= 0.82 | internal |
| Gupta S  2021^31^ | India | NA | NA (66) | Infertility (ART) | Ovarian volume measurement | DL | 3D ultrasound images | 3D Ultrasound Super  Resolution (3DUSSR) network (Sliding window approach with L = 3) | Average SSIM = 0.96 | NA | NA | internal |
| Chen T  2009^32^ | USA | NA | NA (501) | Infertility (ART) | Ovarian follicle segmentation | ML | 3D ultrasound images | Clustered Marginal Space Learning (probabilistic boosting tree) | Missed segmentation ratio (MSR): MSR = 20.5% | NA | NA | internal |
| Li H  2019^33^ | China | NA | 219 (3204) | Infertility (ART) | Ovary and ovarian follicle segmentation | DL | 2D ultrasound images | CR—Unet | for follicles: DICE = 0.86 for ovary: DICE =0.91 | *U-Net  *DeepLabV3+  *PSPNet-1 | U-Net:  For follicles:  DICE = 0.82  For ovary:  DICE = 0.88  DeepLabV3  For follicles:  DICE = 0.79  For ovary:  DICE =0.86  PSPNet-2  For follicles:  DICE =0.82  For ovary:  DICE = 0.87 | internal |
| Carvalho C  2019^34^ | Portugal | NA | NA (107) | Infertility | Ovary and ovarian follicle segmentation | DL | *Ultrasound images and BRF (beam-formed radio-frequency)  *+2D ultrasound images | *U-Net (for ovary)  ** fully connected residual network (FCRN) (for follicles) | *for ovary: DICE = 0.93 **for follicles: DICE = 0.91 | * U-Net (B-mode&BRF (as model’s input) for ovary  ** FCRN (B-mode as model’s input) for follicles | *for ovary:  B-mode BRF + U-Net  DICE = 0.93  **for follicles:  B-mode + FCRN  DICE = 0.89 | internal |
| Robertson I  2021^35^ | UK | 2011-2019 | 1875 (9294) | Infertility (ART) | Prediction of hyper-response (oocyte maturation trigger timing) | ML | Ultrasound and clinical features (age) | Random forest regressor | AUC = 0.91 | NA | NA | internal |
| Srivastava D 2021^36^ | India | NA | 26 (192) | Infertility (ART) | Automated longitudinal tracking of follicular growth | DL | 3D ultrasound images | SFR—Net | DICE = 0.86 | NA | NA | internal |
| Liang X  2020^87^ | China | 2019 | 106 (NA) | Infertility (ART) | Follicle diameter measurement | DL | Ultrasound images | CR-Unet | ICC (intraclass correlation coefficient) = 1 | NA | NA | internal |
| Li ZY,  2024^37^ | China | 2022-2023 | 111 | Infertility | Evaluation of endometrial elasticity measured by Shear wave elastography (SWE) in assessing patients with unexplained infertility | ML | endometrial  mean elasticity (E-mean) | Logistic Regression (LR) | AUC = 0.89 | *LR (shear wave velocity as model’s input)  *LR (endometrial thickness as model’s input)  *LR (uterine artery as model’s input) | *LR (shear wave velocity as model’s input):  AUC = 0.89  *LR ( endometrial thickness as model’s input):  AUC = 0.80  *LR (uterine artery as model’s input):  AUC = 0.76 | no |
| Bones E, 2024^86^ | NA | NA | NA (298 volumes) | Infertility  (general population/infertile woman/women with spontaneous miscarriages) | Automated uterine shape segmentation using 3D ultrasound | DL | Ultrasound images | nnU-net | DICE = 0.90 | *Swin UNETR | Swin UNETR *pretrained*  *DICE = 0.83* | internal |
|  |  |  |  |  |  |  |  |  |  |  |  |  |
| Kumar HP, 2014^38^ | India | NA | NA (210) | PCOS | Discrimination between PCO and normal ovary | DL | Shape and intensity features | Probalistic Neural Network (PNN) | accuracy=0.97 | SVM, RBF | SVM:  accuracy = 0.92  RBF :  accuracy = 0.88 | internal |
| Alamoudi A  2023^39^ | Arabia Saudita | NA | 285 (285) | PCOS | Discrimination between PCO and normal ovary | DL | ultrasound and clinical features (anthropometric data, hormonal status, lipid profile, martial status) | MobileNet | accuracy= 0.82 | *VGG16  *VGG19  *InceptionV3  *DenseNet121  *DenseNet201 | VGG16 :  accuracy = 0.78  (typeii)  VGG_19 :  accuracy= 0.75  Inception v3 :accuracy= 0.79  DenseNet 121: accuracy =0.70  DenseNet 201: accuracy= 0.77 | internal |
| Suha SA  2022^40^ | Bangladesh | NA | NA (594) | PCOS | Discrimination between PCO and normal ovary | DL, ML | DL: ultrasound images  ML: deep features from VGGNet16 | DL: VGGNet16 (CNN network)  ML:  Ensemble Model (LR + SVM+DT+KNN+NB) + eXtreme Gradient Boosting (XGBoost) model as Meta-Learner Model | AUC = 0.999 | *Esemble model + Random Forest meta-learner  *Esemble model + AdaBoost meta-learner  *Esemble model + GradBoost meta-learner  *Esemble model +CatBoost meta-learner | Esemble model + Random Forest meta-learner  AUC = 0.98  Esemble model + AdaBoost meta-learner  AUC = 0.996  Esemble model + GradBoost meta-learner  AUC = 0.99  Esemble model +CatBoost meta-learner  AUC = 0.996 | internal |
| Cahyono B  2017^41^ | NA | NA | NA (54) | PCOS | Discrimination between PCO and normal ovary | DL | Ultrasound images | Convolutional Neural Network Network (CNN) | F1-score: 0.76 | NA | NA | internal |
| Madhumitha J 2021^42^ | NA | NA | NA (NA) | PCOS | Discrimination between PCO and normal ovary | ML | Morphological features | Combination of Logistic regression, kNN, Support Vector Machine (SVM) | accuracy = 0.98 | *SVM (Support Vector Machine)  *KNN (K-Nearest Neighbors)  *LR Logistic  Regression | SVM:  accuracy = 0.92  KNN:  accuracy = 0.97  LR:  accuracy = 0.94 | internal |
| Lawrence M J 2007^43^ | Canada | NA | NA (70) | PCOS | Discrimination between PCO and normal ovary | ML | Stereological features | Linear Discriminant classifier | accuracy = 0.93 | SVM (Support Vector Machine)  algorithm, KNN (K-Nearest Neighbors) algorithm | SVM  accuracy = 0.91  KNN  accuracy = 0.91 | internal |
| Cheng JJ  2019^44^ | USA | 2003-2016 | 25535 (39093) | PCOS | Discrimination between PCO and normal ovary | ML | Ultrasound images | Rule-based classifier (RBC) | accuracy = 0.98 | Gradient boosted trees | Gradient boosted trees  accuracy = 0.97 | internal |
| Sumathi M  2024^45^ | NA | NA | NA (1918) | PCOS | Discrimination between PCO and normal ovary | ML/DL | Statistic and Textural Features | Darknet-19 | Accuracy= 0.99 | *AlexNet,  *SqueezeNet  * SVM (Support Vector Machine) | AlexNet:  accuracy = 0.78  SqueezeNet:  accuracy = 0.98  SVM:  accuracy = 0.84 | internal |
| Jeevitha S  2022^46^ | India | NA | NA (100) | PCOS/ Benign ovarian Cysts | Discrimination between PCO and ovarian cyst and normal ovary | ML | Geometrical features | Support Vector Machine (SVM) | accuracy = 0.94 | NA | NA | internal |
| Gopalakrishnan C 2021^47^ | India | NA | NA (90) | PCOS/ Benign ovarian Cysts | Discrimination between PCO and ovarian cyst and normal ovary | ML | Geometrical features | Support Vector Machine (SVM) | accuracy = 0.94 | *Random forest  *Linear discriminant analysis,  *Naı¨ve bayes | Random forest accuracy =0.90    Linear discriminant analysis  accuracy = 0.91  Naı¨ve bayes accuracy = 0.88 | internal |
| Suganya Y  2022^48^ | India | NA | NA (400) | PCOS/ Benign ovarian Cysts | Discrimination between PCO and ovarian cysts | ML | Shape, statistical and textural features | AdaboostRF (Ensemble of Adaboost and Random Forest) | accuracy= 0.87 | *Random Forest  *KNN  * BaggingKNN | KNN:  accuracy = 0.82  BaggingKNN: accuracy = 0.88  Random Forest: accuracy = 0.86 | internal |
| Kiruthika V  2023^49^ | India | 2018 | 105 (NA) | PCOS/ Benign ovarian Cysts | Discrimination between PCO and ovarian cyst and normal ovary | ML | Intensity, Textural, Demographic and Diagnostic | Support vector machine | Accuracy = 0.98 | *Artificial Neural Network (ANN)  *Discriminant classifier | ANN accuracy = 0.97  LDA accuracy = 0.97 | internal |
| Guerriero S  2021^50^ | Italy | NA | 333 (NA) | Endometriosis | Detection of rectosigmoid endometriosis | DL | Ultrasound and clinical features (age) | Neural Network (NNET-neuralnet) | AUC = 0.82 | *k-nearest neighbors algorithm (k-NN)  *Naive Bayes  *Support Vector Machine (SVM)  *Decision Tree  * Random Forest  *Logistic Regression | k-NN AUC = 0.75  Naive Bayes AUC = 0.81  Support Vector Machine AUC = 0.78  Decision Tree AUC = 0.77  Random Forest AUC = 0.77  Logistic Regression AUC = 0.81 | internal |
| Maicas G  2021^51^ | Australia | 2018-2020 | 749 (749 videos) | Endometriosis | Detection of sliding sign (Douglas obliterated) | DL | Ultrasound videos | ResNet network | AUC = 0.96 | NA | NA | internal |
| Yang M  2021^52^ | China | 2015-2018 | 326 (NA) | Endometriosis | Detection of deep endometriosis | DL | Ultrasound images | VGG-GAP model (visual geometry group - Global average pooling) | accuracy = 0.97 | *DSIFT  * CNN  *VGG-16  *VGG-19  * AlexNet model | DSIFT:  Accuracy=0.81  CNN:  Accuracy=0.86  VGG-16:  Accuracy = 0.87  VGG-19  Accuracy = 0.92  AlexNet mode :  Accuracy = 0.94 | internal |
| Podda AS  2024^53^ | Italy | NA | NA (75) | Endometriosis | Detection of deep endometriosis | DL | transvaginal ultrasound images | DenseNet121 U-net ensemble | DICE = 0.82 | *VGG19 U-net  * Custom U-net | VGG19 U-net ensemble:  DICE = 0.76  Custom U-net ensemble:  DICE = 0.77 | internal |
| Stefan RA  2021^54^ | Romania | 2017-2019 | 56 (NA) | Endometriosis | Discrimination between endometriomas and hemorrhagic ovarian cysts | ML | Textural features | Multiple regression analysis | AUC =1 | NA | NA | no |
| Hu P  2023^55^ | China | 2014-2021 | 202 (NA) | Endometriosis/ Pelvic infection | Discrimination between tubal -ovarian abscess and ovarian endometriosis | DL | Ultrasound images | ResNet-152 | AUC = 0.99 | *DenseNet-161  *EfficientNet-B7 | DenseNet-161 AUC = 0.92  EfficientNet-B7 AUC = 0.98  , | internal |
| Sohail ASM  2010^56^ | Canada | NA | NA (478) | Benign ovarian Cysts | Discrimination between different benign ovarian cysts | ML | Statistical and textural features | Fuzzy KNN | accuracy = 0.88 | * SVM  * Ordinary k- Nearest Neighbor  * Neural Network (NN) | SVM:  Accuracy = 0.87  ordinary k Nearest Neighbor:  Accuracy = 0.83  Neural Network (NN):  Accuracy = 0,75 | internal |
| Narmatha C  2023^57^ | Canada | NA | NA (478) | Benign ovarian Cysts | Discrimination between different ovarian cysts | DL | * ultrasound images  ** CNN features | * Convolutional neural network (CNN), **Deep Q-Network (DQN) -   Harris Hawks Optimization (HHO) classifier | accuracy = 0.97 | *ANN  *CNN  *Alexnet | ANN  Accuracy = 0.93  CNN  Accuracy = 0.94  Alexnet  Accuracy = 0.95 | internal |
| Fan J  2023^58^ | China | NA | NA (750) | Benign ovarian Cysts | Discrimination between different ovarian cysts | DL | ultrasound image | Ocys-Net | Accuracy=0.96 | *MobileNetV1  *MobileNetV2  *MobileNetV3  *EfcientNet  *MixNet  *GhostNet  *ShufenetV2 | MobileNetV1 Accuracy = 0.92  MobileNetV2  Accuracy = 0.94  MobileNetV3  Accuracy = 0.93  EfcientNet  Accuracy = 0.95  MixNet  Accuracy = 0.92  GhostNet  Accuracy = 0.95  ShufenetV2  Accuracy = 0.92 | internal |
| Li Y  2024^59^ | China | 2018-2022 | 406 (1601) | Benign ovarian Cysts | Discrimination and automatic segmentation of ovarian endometriomas | DL | ultrasound image | Hybrid model composed by ResNet50 and ViT | Accuracy = 0,91 | *Resnet18  *Resnet50  *Resnet101  *Densenet  *Efficientnet  *Mobilenet  *ViT  *CrossViT  *DynamicViT  *IITC-Net  *MB-DCNN  *DCN | Resnet18  accuracy= 0.89  Resnet50  Accuracy = 0.89  Resnet101  Accuracy = 0.89  Densenet  Accuracy = 0.87  Efficientnet  Accuracy = 0.89  Mobilenet  Accuracy = 0.90  ViT  Accuracy = 0.85  CrossViT  Accuracy = 0.79  DynamicViT  Accuracy = 0.88  IITC-Net  Accuracy = 0.90  MB-DCNN  Accuracy = 0.90  DCN  Accuracy = 0.91 | internal |
| Liu L  2024^60^ | China | 2021-2023 | 407 (NA) | Benign ovarian Cysts | Discrimination between ovarian endometriomas and ovarian dermoid cysts | ML | Clinical (age and presenting symptoms) features and radiomic features | nomogram using the LightGBM algorithm | AUC 0.99 | Support Vector  Machine (SVM) ( only radiomics features as model’s input)  k-nearest neighbor (KNN) ( only radiomics features as model’s input)  LightGBM ( only radiomics features as model’s input)  Multi-  Layer Perception (MLP)  LASSO Logistic Regression (LR) ( only radiomics features as model’s input) | Support Vector  Machine (SVM) AUC = 0.97  k-nearest neighbor (KNN) AUC = 0.98  LightGBM  AUC = 0.97  Multi-  Layer Perception (MLP)  AUC = 0.98  LASSO Logistic Regression (LR) AUC =0.98 | internal |
| Miao K  2024^61^ | China | 2018-2023 | 184 (786) | Benign ovarian Cysts | Discrimination between ovarian endometriomas and ovarian mucinous cystadenomas | DL | ultrasound images | ConvNeXt algorithm | AUC = 0.90 | NA | NA | internal |
| Yu L  2022^62^ | China | 2019-2021 | 120 (NA) | Premature Ovarian Failure | Diagnosis of idiopathic premature ovarian failure (POF) with automated segmentation | ML | Ultrasound images | Improved Mean Shift Algorithm | DICE = 0.90 | NA | NA | internal |
| Atia O  2023^63^ | Israel | 2014-2021 | 503 (NA) | Adnexal Torsion | Discrimination between adnexal torsion and non-adnexa torsion | ML | Clinical ( pain,  nausea, lack of urinary symptoms) and ultrasound features | Multivariate logistic regression model | AUC = 0.75 | Random Forest | RF:  Accuracy = 0.71 | internal |
| Otjen JP  2020^64^ | USA | 2004-2016 | 430 (NA) | Adnexal Torsion | Discrimination between ovarian torsion and non-torsion. | ML | ultrasound and clinical features  (age) | Random Forest | AUC = 0.99 | Decision tree | DT :  AUC = 0.96 | internal |
| Chen S  2024^65^ | China | 2017-2022 | 326 (NA) | Adnexal Torsion | Prediction of ovarian torsion in pediatric patients | ML | Clinical and laboratory (pain, hematocrit, phlogosis indices) and sonographic features | Logistic Regression | AUC = 0.87 | NA | NA | no |
| Turki A  2023^66^ | Arabia Saudita | 2017-2021 | 41 (NA) | Adnexal Torsion | Prediction of ovarian torsion in pediatric patients | ML | Clinical (  pain duration, white blood cell (WBC) counts) and ultrasound features | SVM linear kernel | AUC = 0,99 | *Logistic regression  *XGBoost  *SVM Polynomial  *SVM Radial Basis | Logistic regression  AUC = 0.97  XGBoost  NA  SVM Polynomial  AUC = 0.95  SVM Radial Basis  AUC = 0.93 | internal |
| Garcia-Mejido JA  2024^67^ | Spain | 2023 | 110 (15,932 frames) | Pelvic floor disorders | Identifying pelvic floor structures | DL | Ultrasound videos | FPN + ResNet50 networks | DICE = 0.79 | UNet and LinkNet networks | NA | internal |
| Rabbat N  2023^68^ | USA | NA | 28 (1015) | Pelvic floor disorders | Automated segmentation of the levator ani muscle from 3D endovaginal ultrasound images | DL | 3D endovaginal ultrasound images | U-Net | DICE = 0.86 | *Attention UNet  *FD-UNet  ^*^Dense-UNet | Attention UNet DICE = 0.85  FD-UNet DICE = 0.84  Dense-UNet DICE = 0.81 | internal |
| Zhu Y  2022^69^ | China | 2018-2020 | 80 (3619) | Pelvic floor disorders (TU-LESS) | Identifying pelvic floor function | DL | Ultrasound images | Bilinear convolutional neural network (BCNN-S) | accuracy = 0.89 | BCNN-R | BCNN-R  accuracy = 0.86 | internal |
| Van den Noort F 2023^70^ | Netherlands | 2018-2019 | 304 (790 frames) | Pelvic floor disorders (POP) | Analysis of the muscle movement present in the 4D Transperineal ultrasound (4D TPUS) | DL, ML | 4D TPUS movies | * DL: 3D-convolutional autoencoder (3D-CEA) network ** ML: Gaussian mixture modeling (GMM)- Principal component analysis (PCA) | accuracy = 0.91 | *K-Means  *GMM  *PCA-Kmeans  *D-CAE – k-means  *D-CAE- GMM | K-Means:  accuracy =0.91  GMM:  accuracy =0.58  PCA-Kmeans:  accuracy =0.91  D-CAE – k-means  accuracy =0.88  D-CAE- GMM:  accuracy =0.84 | internal |
| Muta M  2024^71^ | Japan | 2022 | 56 (1144 videos) | Pelvic floor disorders (POP) | Analysis of pelvic floor muscle contraction using self-performed Transabdominal bladder ultrasound | ML | time-series statistical  features | light gradient boosting machine (LightGBM) | AUC = 0.91 | *Logistic  regression  *Random Forest | Logistic  Regression  AUC = 0.61  Random Forest AUC =0.88 | internal |
| Xu H  2021^72^ | China | 2018-2021 | 244 (NA) | Pelvic floor disorders (POP) | Prediction of pessary type (Ring vs Gellhorn) | ML | Ultrasound and clinical features (age, BMI, parity, menopausal status, hiatal  circumference (HC) and pelvic organ prolapse quantification ( POP-Q) measurement) | Multivariate logistic regression | AUC = 0.81 | NA | NA | internal |
| Duan L  2021^73^ | China | 2018 | 64 (64) | Pelvic floor disorders (POP) | Identification of different types of POP (anterior vs middle vs posterior cavity prolapse) | DL, ML | 3D ultrasound images | DL: Deep CNN (DCNN) | AUC = 0.79 | ML:  *SVM  * Radial basis function (RBF)  * KNN | ML:  SVM  AUC:0.74  Radial basis function (RBF) AUC = 0.69  KNN:  AUC 0.55 | internal |
| Liu W  2022^74^ | China | 2021-2022 | 255 (NA) | Pelvic floor disorders (post-partum SUI) | Identification of postpartum SUI risk | ML | Ultrasound and clinical features (age, parity, vaginal delivery) | Logistic regression | AUC = 0.81 | NA | NA | internal |
| Huang Y L  2007^75^ | China | NA | 48 (NA) | Pelvic floor disorders (SUI) | Discrimination between SUI patients and non-SUI patients | DL | Ultrasound features (corrected version of the angle of the bladder neck (CRABN )and the corrected version of bladder neck movement (CBNM) | Multilayer perception neural network | AUC = 0.94 | *Multilayer perception neural network (RABN original roational angle of the bladder neck as model’s input)  *Multilayer perception neural network (BNM original bladder neck movement as model’s input)  * Multilayer perception neural network (CRABN as model’s input)  * Multilayer perception neural network (CBNM as model’s input) | *Multilayer perception neural network (RABN original roational angle of the bladder neck as model’s input)  AUC = 0.77  *Multilayer perception neural network (BNM original bladder neck movement as model’s input)  AUC = 0.72  * Multilayer perception neural network (CRABN as model’s input)  AUC = 0.83  * Multilayer perception neural network (CBNM as model’s input)  AUC = 0.83 | internal |
| Wang X  2022^76^ | China | 2019-2021 | 85 (85) | Endometrial adhesions | Measurement of endometrial thickness using endometrium segmentation | DL | 3D-ultrasound images | 3D Unet | DICE = 0.91 | NA | NA | internal |
| Wu J  2021^77^ | China | 2019-2020 | 54 (54) | Endometrial adhesions | To improve the diagnosis of intrauterine adhesions using a denoising algorithm | DL | 2D-3D ultrasound images | Extreme learning machine denoising algorithm (ELMDA) | MSE = 0.0022, 0.0039, and 0.0071 when the noise variances were 0.1, 0.5, and 1.0, respectively | *Anisotropic diffusion algorithm (ADA)  * Median filter algorithm (MFA) with wavelet threshold WT-MFA | ADA:  denoising MSE  of ADA were 0.0096, 0.0121, and 0.0139 when the noise  variances were 0.1, 0.5, and 1.0, respectively.  WT-MFA:  denoising MSE  were 0.0032,  0.0087, and 0.0135, respectively. | internal |
| Huo T  2023^78^ | China | 2015-2020 | 667 (3870) | Uterine Fibroids | Discrimination between images with uterine fibroids and without fibroids | DL | Ultrasound (gray scale and color Doppler) images | DCNN network | AUC = 0.95 | NA | NA | internal |
| Dilna KT  2022^79^ | NA | NA | NA (259) | Uterine Fibroids | Discrimination between images with uterine fibroids and without fibroids | DL | Ultrasound images | MBF-CDNN (Monarch Butterfly (MB) Optimization and Fuzzy bounding approach based Convolutional Deep Neural Network) | accuracy = 0.95 | *Butterfly  Optimization Based Convolution Deep Neural Network (MB-CDNN)  *Convolution  Deep Neural Network (CDNN)  *Convolutional Neural Network (CNN) | MB-CDNN accuracy = 0.91  CDNN  accuracy = 0.89  CNN  accuracy = 0.84 | internal |
| Shahzad A  2023^80^ | NA | NA | NA (1057) | Uterine Fibroids | Automated detection of uterine fibroids | DL | ultrasound  images | dual-path deep convolutional neural  network (DPCNN) architecture | Accuracy = 0.998 | *VGG16  * ResNet50  *InceptionV3. | VGG16 accuracy= 0.85  ResNet50  Accuracy = 0.89  InceptionV3  Accuracy = 0.90 | internal |
| Cai P  2024^81^ | China | NA | 871 (1990) | Uterine Fibroids | Automated detection of uterine fibroids | DL | ultrasound images | Hybrid model composed by MobileNetV2 and DCGAN | Accuracy = 0.97 | *SVM  *MBF-CDNN  *VGG16  *ResNet18  *ResNet50  *ResNet101  *MobileNetV1  *MobileNetV2 *Swin_transformer | SVM  Accuracy = 0.93  MBF-CDNN  accuracy= 0.95  VGG16  acuracyc = 0.92  ResNet18  accuracy = 0.87  ResNet50  acuracy = 0.89  ResNet101  accuracy= 0.91  MobileNetV1 accuracy = 0.89  MobileNetV2  accuracy = 0.94  Swin_transformer acuracyc = 0.96 | internal |
| Yang T  2023^82^ | China | NA | 871 (871) | Uterine Fibroids | Automated detection of uterine fibroids | DL, | ultrasound images | EfficientNet-YOLOv3 network | Mean average precision (MAP) = 0.98 | *VGG16  *ResNet50  *SSD  *YOLOv3  *YOLOv5  *YOLOv7  *YOLOv5  *YOLOX  *EfficientDet | VGG16  MAP = 0.92  ResNet50  MAP = 0.92  SSD  MAP = 0.93  YOLOv3  MAP = 0.98  YOLOv5  MAP =0.94  YOLOv7  MAP =0.95  YOLOX  MAP =0.96  EfficientDet  MAP = 0.96 | internal |
| Kaveramma UPP  2023^83^ | India | NA | NA (100) | Uterine Fibroids | Automated detection of uterine fibroids | DL, ML | DL:  Ultrasound Images  ML:  Textural features | DL:  Vision Transformer (ViT) | Accuracy = 0.98 | ML:  *Support Vector Machine (SVM)  *Logistic Regression (LR)  *k-Nearest Neighbor (k-NN) | SVM  accuracy= 0.93  LR  accuracy = 0.84  k-Nearest Neighbor (k-NN) accuracy = 0.76 | internal |
| Chinna LM  2024^84^ | India | NA | NA (1057) | Uterine Fibroids | Automated detection of uterine fibroids | DL | IBSO: Ultrasound images  CRNN: texture, shape, intensity, and spatial relationships features from IBSO + elastography features | IBSO-convolutional recurrent neural network (CRNN) | Accuracy: 0.998 | *SGDInception  V3  *SGD-ResNet50  *SGD-VGG16  * DPCNN | SGDInception  V3 accuracy = 0.90  SGD-ResNet50 accuracy = 0.89  SGD-VGG16 accuracy = 0.85  DPCNN accuracy = 0.998 | internal |
| Raimondo D 2023^85^ | Italy | 2022 | 100 (100) | Adenomyosis | Discrimination between adenomyosis and non-adenomyosis | DL | Ultrasound images | ResNet and Vgg networks | accuracy= 0.51 | NA | NA | internal |

NA=not available. Infertility (ART)=infertile patients undergone Assisted Reproductive Technology (ART). PCOS=PolyCystic Ovary Syndrome. TU-LESS=Transumbilical laparoendoscopic singel-site surgery. SUI=stress urinary incontinence. AUC=Area Under the Curve. POP= Pelvic Organ Prolapse. SSIM=Structural similarity index measure. ML=[Machine learning. DL=Deep Learning.](https://en.wikipedia.org/wiki/Machine_learning) ^§^The model’s input refers to the input of both the best performing model (column “best performing AI model”) and other AI models (column “All other AI models”); if the input of the “all other AI models” differs from the input of the” best performing AI model”, it is specified in parentheses in the “All other AI models”
